# Supplementary material for: Impact of Landscape on Host–Parasite Genetic Diversity and Distribution Using the Puumala orthohantavirus–Bank Vole System
Source: Microorganisms. 2021 Jul 15;9(7):1516. doi: 10.3390/microorganisms9071516 (PMC8306195; doi:10.3390/microorganisms9071516)
Supplement: Supplementary file 1 [file microorganisms-09-01516-s001.zip › microorganisms-1268691-supplementary.pdf]

Supplementary Materials

Table S1. Negative and positive selected sites for the Ardennes Puumala virus (PUUV) strains. SLAC: Single-Likelihood Ancestor Counting; FEL: Fixed-Effects Likelihood; MEME: Mixed Effects Model of Evolution; FUBAR: Fast Unbiased Approximate Bayesian; aBSREL: adaptative Branch-Site Random Effects Likelihood; BUSTED: Branch-site Unrestricted Statistical Test for Episodic Diversification.

	**Evolutionary test**	**S segment**	**M segment**	**L segment**
No. of unique sequences		30	14	12
No. of codons analyzed		433	151	137
No. of codons under negative/purifying selection	FEL	79*	35*	29*
	SLAC	18*	3*	2*
	FUBAR	153**	37**	24**
No. of codons under positive/diversifying selection	FEL	0*	0*	0*
	SLAC	0*	0*	0*
	MEME	0*	0*	0*
	FUBAR	1**	0**	0**
	aBSREL	0***<br>(57 branches tested)	0***<br>(25 branches tested)	0***<br>(21 branches tested)
	BUSTED	0****	0****	0****

* *p*-value = 0.1; ** posterior probability = 0.9; *** *p*-value ≤ 0.05; *p*-value ≥ 0.05.

Table S2. Puumala virus (PUUV) prevalence and genetic diversity of vole populations in the ten sites from the French Ardennes.

**Sites**	**N (Trapping year)**	**PUUV prev. (%)**	**A**	H_0	H_E	F_{IS}	F_{ST}
Hargnies	34 (2008)	29.4	11.38	0.858	0.898	0.046 *	0.011
Woiries	37 (2008)	8.1	10.29	0.847	0.875	0.032 *	0.025
Renwez	38 (2008)	13.2	10.57	0.857	0.876	0.022	0.024
Cliron	34 (2008)	5.9	09.82	0.785	0.866	0.093 *	0.046
Elan	27 (2008)	7.4	10.48	0.833	0.879	0.053 *	0.025
Cassine	27 (2008)	18.5	10.55	0.823	0.883	0.069 *	0.020
Sauville	47 (2008)	0	10.44	0.832	0.867	0.052 *	0.045
Croix-aux-Bois	31 (2008)	9.7	11.05	0.840	0.885	0.040 *	0.019
Boult-aux-Bois	16 (2010)	12.5	09.09	0.850	0.845	-0.006	0.056
Briquenay	22 (2008)	0	09.77	0.837	0.877	0.047 *	0.027

N = number of bank voles trapped; A = allelic richness corrected for sample size; H_0 = average heterozygosity; H_E = average expected heterozygosity; F_{IS} = the genetic isolation estimated by local F_{ST} values; F_{ST} = the degree of gene differentiation among populations in terms of allele frequencies. * significant departures from Hardy-Weinberg equilibrium.

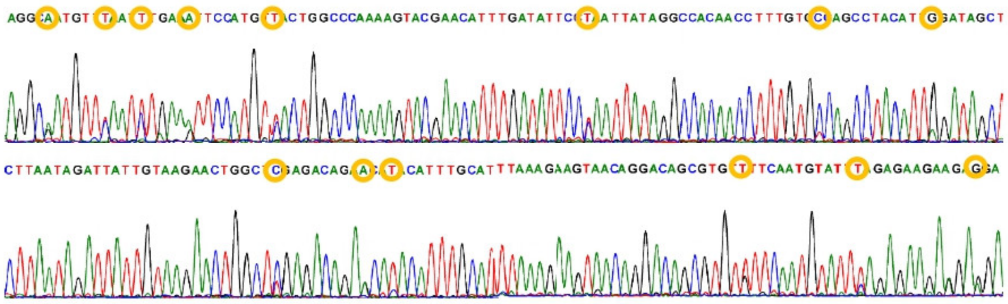

Figure S1. Chromatogram of the Woiries_58 Puumala virus (PUUV) strain. Picture shows nucleotides from 659 to 860 nt of the L genome segment.

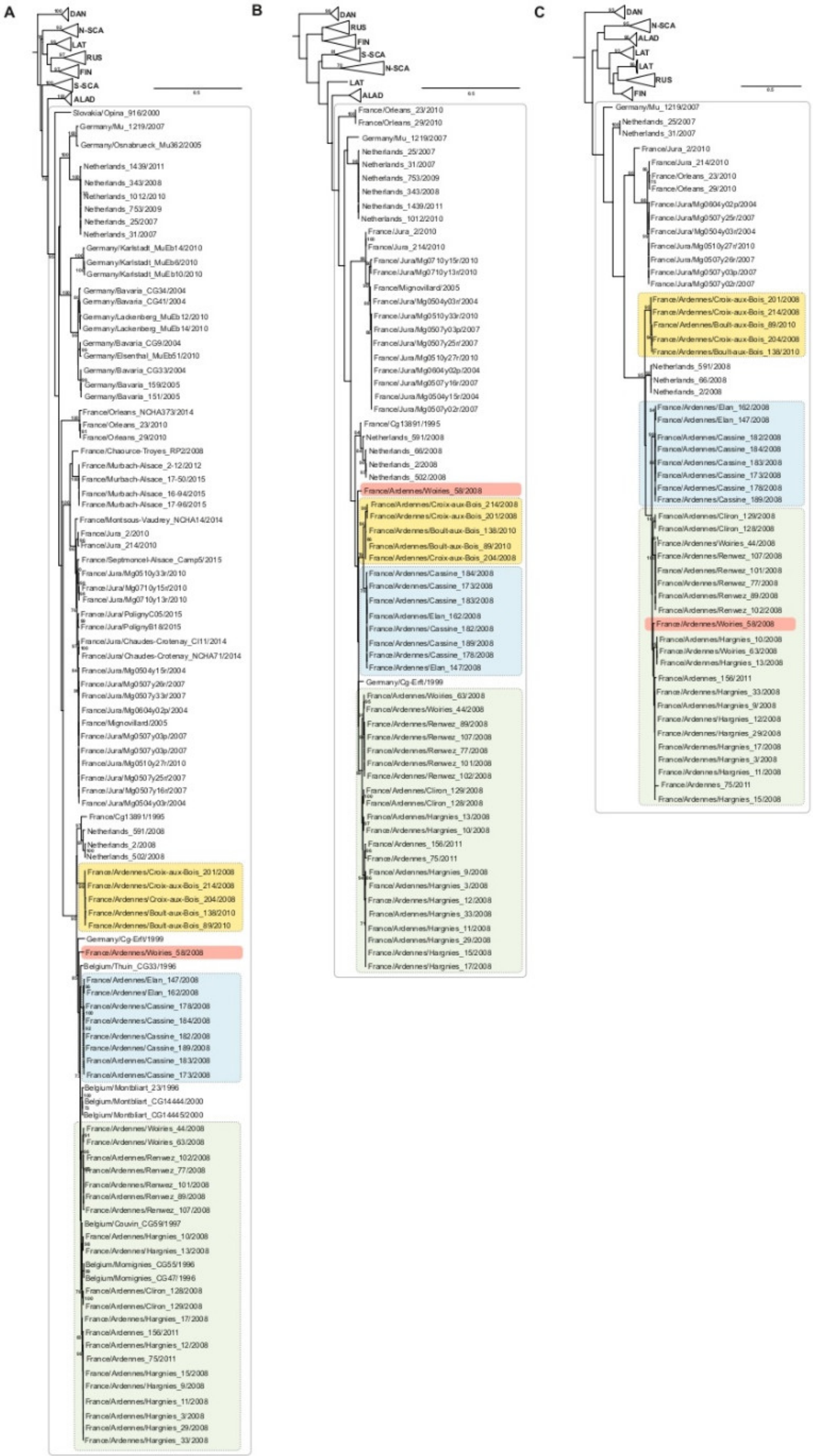

Figure S2. Phylogenetic trees. Maximum likelihood and Bayesian trees calculated for the S, M and L segment sequences of PUUV: (**A**) partial S (1–1299 nt); (**B**) partial M (2180–2632 nt); (**C**) partial L (577–987 nt). A maximum clade credibility trees are presented with nonparametric bootstrap support values (> 70%) shown on nodes. The strains HNTV, ANDV, SNV and TULV were used as outgroups, nonetheless, for the simplification of the graphic they are not included in this figure.
